# Analysing the Renal Vasculature Using Super-Resolution Ultrasound Imaging: Considerations for Clinical and Research Applications

**DOI:** 10.3390/diagnostics15121515

**Published:** 2025-06-14

**Authors:** Amy McDermott, Nathalie Sarup Panduro, Iman Taghavi, Hans Martin Kjer, Stinne Byrholdt Søgaard, Michael Bachmann Nielsen, Jørgen Arendt Jensen, Charlotte Mehlin Sørensen

**Affiliations:** 1 Department of Biomedical Sciences, University of Copenhagen, 2200 Copenhagen, Denmarkcmehlin@sund.ku.dk (C.M.S.); 2 Department of Diagnostic Radiology, Rigshospitalet, 2100 Copenhagen, Denmark; mbn@dadlnet.dk; 3 Center for Fast Ultrasound Imaging, Department of Health Technology, Technical University of Denmark, 2800 Lyngby, Denmarkjaje@dtu.dk (J.A.J.); 4 Department of Applied Mathematics and Computer Science, Technical University of Denmark, 2800 Lyngby, Denmark; hmkj@dtu.dk; 5 Department of Clinical Medicine, University of Copenhagen, 2200 Copenhagen, Denmark

**Keywords:** super-resolution ultrasound, SRUS, ultrasound localisation microscopy, ULM, micro-CT, µCT, microbubbles, SonoVue, Microfil, Zucker rat

## Abstract

**Background:** Vascular imaging is essential for clinical practice, research, and the diagnosis and management of vascular diseases. Super-resolution ultrasound (SRUS) imaging is an emerging high-resolution imaging technique with broad applications in soft tissue vascular imaging. However, the impact of biological and clinical variables on its imaging accuracy is currently unknown. This study investigates these factors in an animal model and compares SRUS with contrast-enhanced µCT. **Methods**: Kidney scans from 29 Zucker rats (Zucker Diabetic Fatty and Zucker Lean) were retrospectively analysed. The left kidney was imaged in vivo using SRUS during microbubble infusion, then filled with Microfil and excised for ex vivo µCT. SRUS parameters and clinical variables were analysed, and SRUS scans were co-registered with µCT to compare vascular density measurements. **Results**: Mean arterial blood pressure and anaesthesia time showed significant linear relationships with SRUS microbubble detection and vascular track reconstruction. The anaesthesia time was also strongly correlated with vascular density measurement. Visualisation and velocity estimations of renal arteries were limited with SRUS. Ultrasound signal attenuation had significant impacts, particularly in cortical far-field imaging. Despite differences between kidney regions, the vascular density distribution did not differ considerably between SRUS and µCT datasets for whole-kidney imaging. **Conclusions**: This study outlines key factors SRUS users must consider for optimal technique use. Careful region selection and control of clinical variables ensure more reliable and comparable images. Further research is necessary to translate these findings from a rat model into clinical application.

## 1. Introduction

Super-resolution ultrasound (SRUS) imaging is a developing medical imaging technology with the potential to improve patient-side vascular imaging resolution significantly. However, the technique has several intrinsic limitations, and its susceptibility to patient and clinical variability has not been fully characterised. This study retrospectively analyses a varied dataset of SRUS rat kidney images, assessing the impact of clinical variables on SRUS image quality. It also presents a novel quantitative vascular density analysis method using SRUS imaging, comparing it with an established technique, ex vivo µCT. These data will give an overview of the intrinsic limitations in SRUS imaging, giving researchers and clinicians valuable insights to guide method selection and minimise variation in SRUS images.

SRUS was inspired by developments in photoactivated localisation microscopy, overcoming the diffraction limit by distinguishing the centre points of two overlapping signal echoes [1,2]. SRUS is a technique which detects, localises, isolates, and tracks individual intravascular microbubbles (MBs) [3]. This method, also known as ultrasound localisation microscopy, generates images of the vascular system by overlaying all detected MB movement trajectories over a given acquisition period [4]. SRUS has been successfully used in experimental settings to visualise various human and animal tissues, with potential imaging resolutions estimated as low as ≈10 µm; in renal imaging, spatial resolutions down to 32 µm have been demonstrated [5,6,7,8,9,10,11].

The challenges limiting SRUS’s practical use are both clinical and technical [12]. The technique requires an intravascular injection with MBs, administered either as a bolus or a continuous infusion [13]. Ultrasound settings must balance clinical needs and MB preservation, with high acoustic pressures potentially causing MB destruction [14,15]. The ideal MB concentration varies by tissue type, requiring optimisation to ensure precise imaging [5,16]. Additionally, selecting the ideal acquisition time is crucial; longer times improve detection but may increase motion artefacts and patient discomfort [5]. SRUS requires high-frame-rate ultrasound systems and advanced computational data post-processing, which are not always available in hospitals and make real-time imaging challenging [17]. Motion artefacts from the operator, patient, or tissue further complicate accuracy. Ongoing research focuses on enhancing reliability by improving MB tracking, imaging frame rates, and deep learning [18,19]. Despite advancements, individual physiological variations and clinical factors affect SRUS accuracy and repeatability, posing ongoing challenges for its widespread clinical use.

Our first aim in this paper is to present a novel assessment of individual and clinical variables’ impact on SRUS vascular reconstruction. We selected the kidney as our imaging target due to its highly vascular nature, the complex vascular network of varying sizes, and its clinical relevance for vascular research. The rat kidney contains a vascular system with vessels of varying diameters: from the renal veins and arteries (100–1300 µm) to the afferent and efferent arterioles (10–20 µm) and the vasa recta capillaries (7–20 µm) [20,21,22]. We retrospectively analyse a large dataset of SRUS rat kidney images, exploring variations in image quality and identifying controllable factors to optimise the technique.

Another key challenge limiting the translation of SRUS into clinical and biomedical research settings is the difficulty of extracting quantitative, clinically relevant data from SRUS images. Consequently, the second aim of this study is to present an SRUS vascular density analysis method. As no non-invasive in vivo imaging techniques can achieve a comparable resolution, micro-computed tomography (µCT) with intravascular contrast is a suitable comparator for assessing SRUS data.

## 2. Materials and Methods

### 2.1. Ethical Considerations

The experiments were conducted at the University of Copenhagen, Department of Biomedical Sciences, under experimental licence number 2020-15-0201-00547, as agreed with the Danish Animal Experiments Inspectorate. All procedures complied with relevant regulations and were performed in accordance with the ARRIVE guidelines.

### 2.2. Animal Population

This exploratory study re-uses data initially collected and published by Søgaard et al., 2023; full experimental methods can be found in that paper, including the collection of data not used here [23]. The original dataset examined twenty-two male Zucker Diabetic Fatty rats (strain 370, obese fa/fa) and twenty-two male Zucker Lean rats (strain 380, Lean fa/+) of varying ages, between 12 and ≈40 weeks old (Charles River Laboratories^©^, Lyon, France). All animals were housed in the Department of Experimental Medicine animal facility at the University of Copenhagen. Animals were fed an ad lib diet of Purina 5008 (LabDiet, St. Louis, MO, USA), had free access to water, and were kept in stacked cages with a maximum of three individuals per cage. This study used a subset of the original population, selecting those where consistent vascular filling with contrast agent was observed on µCT, resulting in a total of 29 rats being included in this study.

### 2.3. SRUS Data Acquisition and Experimental Variables

After anaesthesia and laparotomy, the left kidney was scanned directly with a modified BK5000 scanner and an X18L5s hockey stick transducer (BK Medical, Herlev, Denmark) during a continuous infusion of diluted SonoVue (1:10) (Bracco, Milan, Italy). Full details of animal preparation, laparotomy, in vivo SRUS parameters, research aims, and image reconstruction information are described in Søgaard et al., 2023 [23]. The anaesthesia time was measured from induction until the ultrasound scanning was completed.

#### 2.3.1. MB Detections and MB Tracks

The total number of MB detections and the average number of MB detections per imaging frame were recorded. Average MB detections per imaging frame were calculated as the total number of MB detections divided by the total number of imaging frames, which is the product of the acquisition time and the frame rate of 54 Hz. The number of MB tracks generated with a hierarchical Kalman tracker for each SRUS image was recorded [7]. An MB must be detected and tracked through at least three imaging frames for a track to be reconstructed.

#### 2.3.2. Physiological Parameters

All SRUS scans had a data acquisition time of 10 min. Mean arterial blood pressure (MAP) was continuously recorded during the acquisition period using a Statham P23-dB pressure transducer (Gould, Oxnard, CA, USA) with a catheter placed in the left carotid artery. The average MAP across the 10 min SRUS acquisition time was calculated using LabChart (ADInstruments, Colorado Springs, CO, USA). The LabChart cyclic measurement module was used to extract heart rate (HR) in beats per minute over the acquisition time. All animals were maintained under general anaesthesia using a mechanical ventilator at a set rate of 69 breaths per minute. Consequently, the effect of respiration was not investigated in this study.

#### 2.3.3. MB Flow Velocities

MB flow velocities were measured automatically from SRUS acquisition data. The method involved tracking the velocity of microbubbles by calculating the time derivation of their trajectory locations, as outlined by Taghavi et al., 2021 [24]. The ability of SRUS to detect MBs is known to be limited by the imaging frame rate [19]. To assess how this limit impacts our dataset, the velocity of MB flow in four vessel types was measured: segmental veins, segmental arteries, arcuate veins, and arcuate arteries. Anatomical specialists manually identified the vessel type within the SRUS images. Three different vessels of each type were manually selected from each scan for MB velocity measurement. Preference was given to well-imaged vessels, ideally located in different regions of the kidney. The velocities were averaged to give the average per scan. The average velocity analysis excluded SRUS images containing less than three vessels of a specific type.

### 2.4. Ex Vivo µCT

After the SRUS data acquisition, the left kidneys were infused with the contrast agent Microfil (MV122, Flow Tech Inc., Carver, MA, USA), excised, and scanned ex vivo for 11 h in a ZEISS XRadia 410 Versa µCT scanner (Carl Zeiss Microscopy GmbH, Jena, Germany) with an isotropic voxel size ranging from 22.6 µm to 26.5 µm. The full details of the µCT scans and preparation of the kidneys, including euthanasia of the rats, are presented in Søgaard et al., 2023 [23].

### 2.5. Image Segmentation and Co-Registration

#### 2.5.1. Identification and Isolation of Co-Registered μCT Image Planes

**Figure 1:**
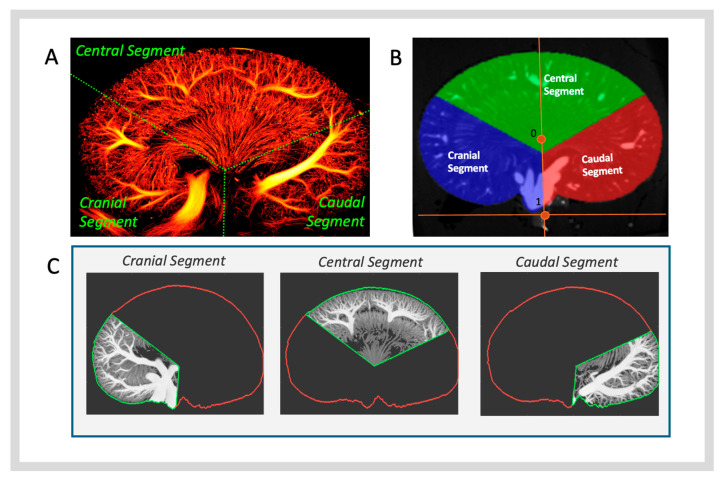
**An overview of the co-registration process between 2D SRUS and 3D µCT.** (**A**) Visual segmentation of the SRUS image into three smaller anatomical segments for co-registration, the cranial, central, and caudal segments. (**B**) Division of the kidney into three 120° segments. The labelled point 0 represents the kidney’s centre of mass; point 1 indicates the shortest distance between point 0 and the long axis of the bounding box. (**C**) Co-registered 2D μCT images post-division and -identification of the co-registered region.

To directly compare two-dimensional (2D) SRUS and three-dimensional (3D) μCT modalities, the same kidney region imaged by 2D SRUS within the 3D μCT volume was identified and isolated. The software ITK-SNAP Version 4.0.1 was utilised [25], allowing a visual overlay of the 2D SRUS image within the 3D μCT volume. Co-registration between 2D SRUS and 3D μCT was achieved by manipulating the SRUS image with translation, rotation, and scaling. Initially, the intention was to manually co-register the complete 2D SRUS image to the closest matching μCT plane using ITK-SNAP; however, due to differences in the scanning plane of the two modalities, full alignment of the SRUS image was not possible [25]. Three co-registrations were created per kidney to overcome this, focusing on smaller anatomical segments: the cranial, central, and caudal segments (Figure 1A). Co-registration of smaller regions enabled the highest degree of co-registration agreement between 2D SRUS and 3D μCT. This process also enabled us to overcome some of the distortions in anatomical accuracy introduced during both the preparation of the kidney for µCT and the SRUS reconstruction.

#### 2.5.2. Anatomical Regions of Interest

During μCT reconstruction, the coronal view was set to display the kidney in the anterior-to-posterior orientation, with alignment based on the tomographic slice showing the organ’s largest cross-sectional area. Three-dimensional segmentation masks were manually generated using the polygon and label interpolation tool (for every 3–5 slices) in ITK-SNAP [25]. A 3D segmentation mask of the full kidney volume was created. The kidney volume mask was then divided into three segments of interest with the following workflow using MATLAB R2021b (The MathWorks Inc., 2022, Natick, MA, USA) (Figure 1B). Using all the voxel coordinates of the kidney mask, a principal component analysis was applied to determine a reference 3D bounding box around the kidney. Subsequently, the centre of the bounding box was identified and designated as point 0. Point 1 was designated as the shortest distance from point 0 to the long axis of the bounding box, specifically toward the renal hilus. A division point was determined to partition the kidney into three segments of interest. From point 0, a line was drawn, following the “axial” direction of the bounding box towards the renal hilus. The division point was placed by a 20% shift in point 0 towards point 1. This 20% shift was implemented to ensure that a larger portion of the kidney located within the ultrasound near field was included in the central segment. From this division point, the image plane was divided into three equal 120° segments, using the axis between point 0 and point 1 as the boundary between the cranial and caudal segments. The kidney 3D volume was fully divided by projecting these three segments through the longitudinal axis. Using the matching co-registration for the SRUS segment and the extracted µCT segment, made it possible to identify the µCT imaging region (Figure 1A,C). The μCT image planes of interest were extracted as a maximum intensity projection across a 2 mm wide region, centred, spaced, and oriented according to the co-registered SRUS image. The slice thickness of 2 mm was chosen to approximately match the “average” elevation depth/thickness of the ultrasound transducer. The 2D μCT images were then re-sampled to 22.6 µm pixel size to match SRUS images (Figure 1C).

**Figure 2:**
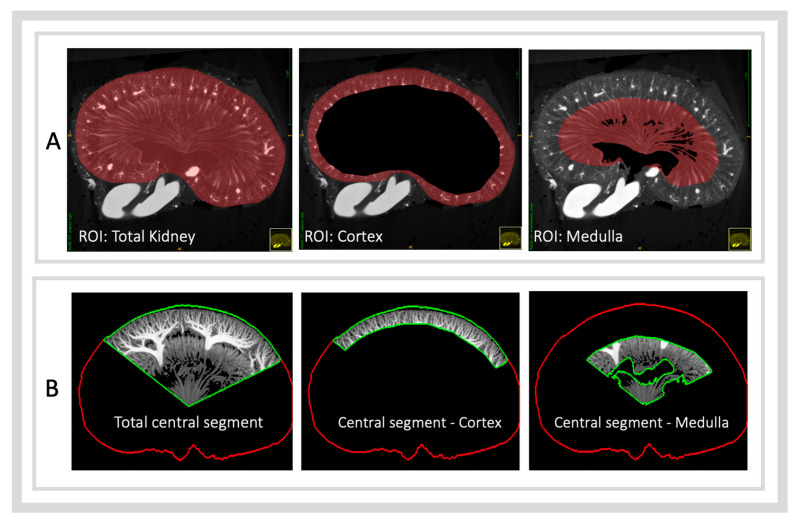
**A visual guide to the anatomical regions analysed** (**A**) **and how they are used to subdivide the kidney segments** (**B**). Image series (**A**) displays the difference between the 3D volume masks on a consistent coronal imaging plane. Image series (**B**) demonstrates the subdivision of the central kidney segment into the cortex and medulla regions.

After creating a µCT segmentation mask of the total kidney volume as a region of interest (ROI), two additional ROIs were isolated from within the total kidney volume: the cortex and the medulla. These ROIs were isolated using a total of four µCT 3D segmentation masks: (1) the total kidney (as previously described), (2) the boundary of the arcuate vessels, (3) the boundary of the vasa recta, and (4) the pelvic cavity. In this study, three anatomical ROIs were analysed and created by subtracting specific segmentation masks as follows (Figure 2A):

- ROI—Total Kidney: Derived directly from the kidney segmentation mask.
- ROI—Cortex: Created by subtracting the arcuate artery boundary mask from the kidney mask.
- ROI—Medulla: Created by subtracting the pelvic mask from the vasa recta boundary mask.

### 2.6. Quantifying Vascular Density

#### 2.6.1. µCT Post-Processing and Vascular Density Assessment

**Figure 3:**
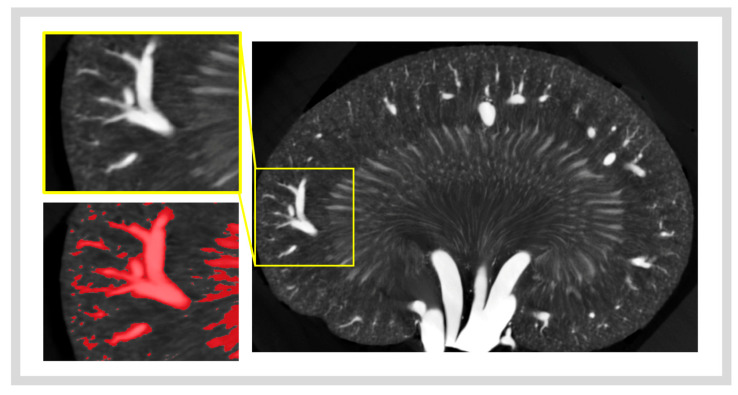
**An image of the vascular segmentation process using intensity thresholding**. The contrast-filled vasculature is selected (highlighted in red) using intensity thresholding, aiming to select the contrast without over-segmentation.

Segmentation thresholds to isolate the contrast-filled vasculature were identified manually for each µCT scan, based on the best isolation of vasculature whilst limiting the inclusion of non-vascular tissues (Figure 3). These thresholds were determined by two blinded individuals trained in clinically assessing µCT images: a medical doctor and a veterinarian. The average of their threshold values was used (low inter-rater variability was present, with an intraclass correlation coefficient of 0.83). Vascular density was calculated as the volume of segmented vascular tissue divided by the total volume of the area of interest. These regions were then isolated within the co-registered μCT image plane and exported as separate 2D images (Figure 2B).

#### 2.6.2. SRUS Post-Processing and Vascular Density Assessment

SRUS motion correction, MB detection, isolation, localisation, tracking, and vascular reconstruction were completed in MATLAB R2021b (The MathWorks Inc.), following the methods described by Taghavi et al., 2021 [26]. Image reconstruction used a non-rigid motion correction based on speckle tracking, as outlined in the same study.

A threshold for vascular track inclusion was set at >1 MB detection per pixel over the total scanning time. To make the processing less dependent on pixel size and dynamic range, MB tracks were given an estimated diameter based on the resolution of the SRUS scanning system. This resolution was estimated using the average Fourier ring correlation on a total of 96 SRUS scans from the 44 rat kidneys with the same SRUS system and settings, resulting in a mean resolution and MB track diameter of 42.25 μm (ranging from 20 to 70 μm). Then, the image was re-sampled to a 22.6 μm pixel size to match the lowest resolution of the μCT scans. Each SRUS image was split into the three kidney segments of interest and further subdivided based on the anatomical region of interest (see Section 2.5.2). SRUS vascular density was calculated on this binary image within a specified region of interest.

### 2.7. Statistical Analysis

The statistical significance threshold was set at *p* < 0.05 throughout this study. Results are marked with one asterisk (*) for *p* < 0.05, two asterisks (**) for *p* < 0.01, and three asterisks (***) for *p* < 0.001. An analysis of variance (ANOVA) was used to compare the means of different groups. When controlling for covariates, specifically age in this study, an analysis of covariance (ANCOVA) was applied. Tukey’s post hoc test was conducted following significant ANOVA results to perform pairwise comparisons between the group means. Tukey’s test was selected instead of Bonferroni’s as this is an exploratory study with similar group sizes. A two-way repeated-measures ANOVA was used to analyse SRUS and µCT vascular density data for different anatomical regions. Pearson’s correlation was used to investigate the linear relationship between two continuous variables. A correlation matrix was used to display the correlation coefficients between multiple variables to examine the effect of clinical variables on SRUS image reconstruction parameters. Multiple linear regression was applied to investigate the relationship between MAP and anaesthesia duration with SRUS quantitative parameters. The Kolmogorov–Smirnov test was used to analyse the distribution of density data for SRUS and µCT. For boxplots, the box represents the interquartile range, the horizontal line indicates the median and the whiskers represent the range of the data, excluding outliers. Outliers were defined as any data points falling outside 1.5 times the interquartile range. All statistical analyses and graphical presentations were performed using R version 4.3.2 (R Core Team, 2024, Vienna, Austria).

## 3. Results

### 3.1. The Impact of Experimental Variables on SRUS Imaging

**Figure 4:**
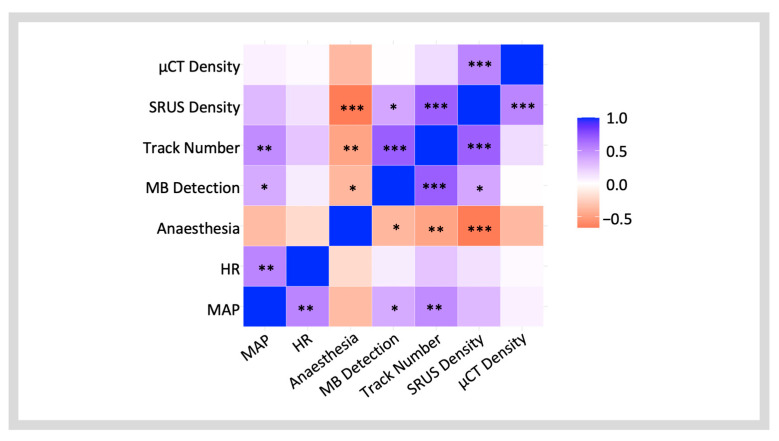
**A correlation matrix showing the linear relationships between independent experimental factors (blood pressure, heart rate, and anaesthesia time), the SRUS technical parameters, and the vascular density measurements from SRUS and µCT**. The *r*-value is shown using the colour dynamic range from blue to red. The appropriate asterisks show the *p*-value, one asterisk (*) for *p* < 0.05, two asterisks (**) for *p* < 0.01, and three asterisks (***) for *p* < 0.001.

A correlation matrix was used to characterise the inter-dependent relationships between independent experimental factors (blood pressure, heart rate and anaesthesia time) and the SRUS and µCT quantitative parameters: MB detection rate, the number of reconstructed tracks, and density measurements (Figure 4).

#### 3.1.1. Blood Pressure and Heart Rate

**Figure 5:**
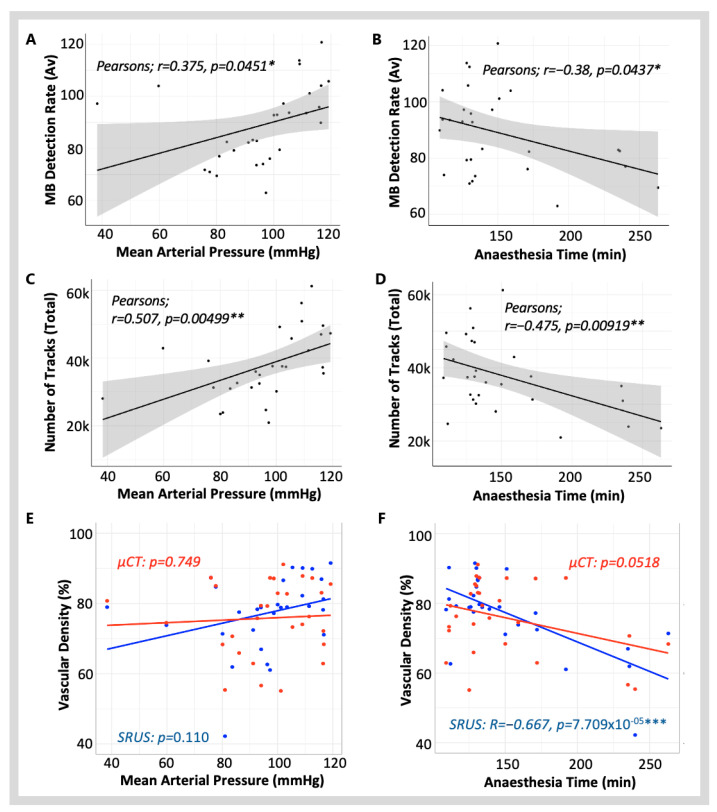
**Scatter plots illustrating the impact of mean arterial pressure (MAP)** (**A,C,E**) **and anaesthesia time** (**B,D,F**) **on SRUS technical parameters.** Panels (**A**,**B**) show the microbubble (MB) detection rate, (**C**,**D**) depict the total number of vascular tracks reconstructed, and (**E**,**F**) present measured vascular density. In graphs (**E**,**F**), vascular density measurements from SRUS (blue) and µCT (red) are overlaid on the same axis for direct comparison. The appropriate asterisks show the *p*-value, one asterisk (*) for *p* < 0.05, two asterisks (**) for *p* < 0.01, and three asterisks (***) for *p* < 0.001.

At the time of scanning, the MAP ranged from 38 mmHg to 119 mmHg, and the HR ranged from 84 BPM to 346 BPM. Variation in these cardiac parameters at the time of scanning can be caused by using a vasoactive agent, bleeding from surgical incisions, and the differing health status of the animals. Additionally, the population included animals with hypertensive conditions associated with diabetes and healthy animals, introducing further variability. HR had a significant, positive linear correlation with MAP (Pearson’s; *r* = 0.5324, *p* = 0.00294 **) but no significant linear relationship to any other variable measured in this study. Conversely, the MAP showed significant linear relationships with both SRUS technical parameters. There was a significant linear correlation between MAP and MB detection rate (Pearson’s; *r* = 0.375, *p* = 0.0451 *) (Figure 5A) and MAP and the number of tracks (Pearson’s; *r* = 0.507, *p* = 0.00499 **) (Figure 5C). While these data show that a higher MAP is associated with an increase in MB detections and a greater number of MB tracks, no significant correlation was observed between MAP and SRUS vascular density (*p* = 0.110) (Figure 5E).

#### 3.1.2. Anaesthesia Time

Due to experimental factors, the time the animal spent under anaesthesia before SRUS scanning varied between 109 min and 263 min. This introduced variation in the time of isoflurane exposure and length of surgery. The length of anaesthesia before scanning had a significant linear correlation to the MB detection rate (Pearson’s; *r =* −0.38, *p* = 0.0437 *) (Figure 5B), the number of MB tracks (Pearson’s; *r* = −0.475, *p* = 0.00919 **) (Figure 5D) and the SRUS vascular density measurements (Pearson’s; *r* = −0.667, *p* = 7.709 × 10^−5^ ***), with longer anaesthesia times causing a significantly lower measured SRUS vascular density. No statistically significant linear relationship existed between anaesthesia time and vascular density when measured from µCT (*p* = 0.0518) (Figure 5F).

As there is a known causal relationship between isoflurane anaesthesia and hypotension [27], a multiple linear regression model was used to identify whether MAP exacerbates the effect on SRUS density. The model showed that, together, the MAP and anaesthesia time account for a significant amount (*F*(3,25) = 6.94,  *p* = 0.00150 **) of the variability in SRUS vascular density, explaining 45.44% of the variance (*R*^2^ = 0.454, Adjusted *R*^2^ = 0.389). This model detected no significant main effect with MAP (*p* = 0.705) or anaesthesia time (*p* = 0.372), and no significant interaction was seen between them (*p* = 0.648). Expanding the model to include HR and its interactions yielded a similar level of explanatory power (*R*^2^ = 0.47), but with a reduced adjusted *R*^2^ of 0.29. This full model also reached statistical significance (*F*(7,21) = 2.66, *p* = 0.039 *), yet no individual variable or interaction term reached significance (*p* > 0.05). The inclusion of HR did not improve the model substantially, suggesting HR does not independently affect SRUS vascular density in this data.

#### 3.1.3. Other Clinical Variables

The study population contains a high degree of individual physical variation, including sick animals (varying severity of diabetes) and healthy animals of various ages. The differences in health status are investigated in Søgaard et al., 2023 [23].

To examine the impact of age on MB detection, an ANOVA was conducted within the healthy rat sub-population (Zucker Lean rats) to examine the impact of age on MB detection. This sub-population was selected because the vascular density, measured with µCT, did not change with age (ANOVA; *p* = 0.158). In this population, age had a statistically significant association with the MB detection rate (*F* = 3.861, *p* = 0.0483 *), with a general trend between increasing age and higher average MB detection rates. Tukey’s honest significant difference test identified a significant pairwise comparison between age 12 and 40 weeks (*p* = 0.0430 *); no other pairwise comparisons were significant. Age also had a significant effect on total track number (*F* = 4.636, *p* = 0.0302 *); however, there were no significant pairwise effects. Age had no significant effect on the final SRUS density (*p* = 0.533).

As blood pressure affects MB detection, an ANCOVA was used to examine whether the significant effect of age might be due to age-associated changes in blood pressure. The main effects of age (*F*(2,10) = 5.95, *p* = 0.0199 *) and blood pressure (*F*(1,10) = 6.61, *p* = 0.0279 *) in isolation significantly influenced average MB detection, but there was no significant interaction between the two (*F*(2,10) = 1.71, *p* = 0.230).

Kidney size and vascular density vary between individuals. We investigated whether these variables may impact SRUS data collection. The kidney size (volume) increases from 12 to 40 weeks old rats, as measured using the µCT. There was no linear relationship between the kidney size and the MB detection rate per imaging frame (Pearson’s; *r*= −0.187, *p* = 0.331). The vascular density of the imaging plane, quantified from the µCT, also varied across the population. The Pearson’s correlation showed that this had no detectable linear relationship with the average MB detection (Pearson’s; *r* = −0.00825, *p* = 0.966).

### 3.2. Imaging Blood Vessels with Varying Blood Flow Velocities

The MB flow velocity is a main factor affecting the MB detection rate, as all SRUS systems have a velocity detection limit due to the imaging frame rate. A frame rate of 54 Hz was used in this study, which resulted in an inability to detect MBs moving faster than 15 mm/s.

**Figure 6:**
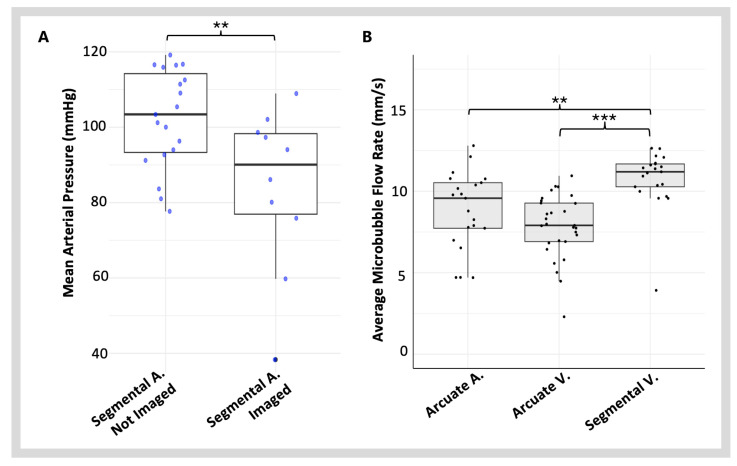
**MB flow dynamics in the renal vascular system.** (**A**) The impact of mean arterial blood pressure during scanning on imaging segmental arteries with SRUS. (**B**) The variation in MB flow velocities in different vessel types within the renal vasculature. The appropriate asterisks show the *p*-value, two asterisks (**) for *p* < 0.01, and three asterisks (***) for *p* < 0.001.

To characterise how this limitation impacts image construction using a renal dataset, where there are large variations in vessel size and expected blood flow velocity, four vessel types were selected: segmental arteries, arcuate arteries, segmental veins, and arcuate veins. Segmental veins and arcuate veins were imaged in all scans. Detection of the arterial system was less consistent, with only 34% of scans containing at least one segmental artery and 90% of scans containing at least one arcuate artery. To analyse whether a decrease in blood pressure could increase the detection of MBs within the arterial system, the individuals were identified where at least one segmental artery was imaged. A t-test revealed a statistically significant difference in MAP (*t* = 2.84, *df* = 27, *p* = 0.008 **) between individuals with imaged segmental arteries (MAP = 84 mmHg ± 21.6) and those without (MAP = 102 mmHg ± 13.1) (Figure 6A). Further analysis of the average MB velocity of the segmental arteries could not be completed as no scan included three separate vessels of this type.

An ANOVA was used to analyse the MB flow velocity between the three other vessel types. The vessel type had a significant association with MB velocities (*F*(2,76) = 18.2, *p* = 3.48 × 10^−7^ ***), and the pattern between vessel types did not match the biologically expected pattern of faster velocity in the arterial system (Figure 6B). The average MB flow velocity was fastest in the large segmental vein and significantly faster than those detected in the arcuate arteries (*p* = 0.0010 **) and the arcuate veins (*p* = 2.00 × 10^−7^ ***). A linear correlation analysis identified how the MAP affected the MB flow velocity in these different vessels. The MAP had a significant linear correlation with the MB flow velocities in both the segmental veins (Pearson’s; *r* = 0.458, *p* = 0.0125 *) and arcuate veins (Pearson’s; *r* = 0.579, *p* = 9.93 × 10^−4^ ***) but not in the arcuate arteries (*p* = 0.540).

### 3.3. Vascular Density

#### 3.3.1. Regional Variations in Vascular Density Measured with SRUS

MB detection and vascular track reconstructions can be considered raw data collection points, enabling SRUS vascular density measurements. A significant, positive correlation existed between MB detection and the number of tracks (*r*(27) = 0.691, *p* = 3.37 × 10^−5^ ***) and the SRUS vascular density measurement (*r*(27) = 0.393, *p* = 0.0348 *) (Figure 4). However, the correlation between MB detection and vascular density was weaker. In contrast, a stronger positive correlation was seen between the number of tracks and the SRUS density measurement (*r*(27) = 0.683, *p* = 4.52 × 10^−5^ ***).

**Figure 7:**
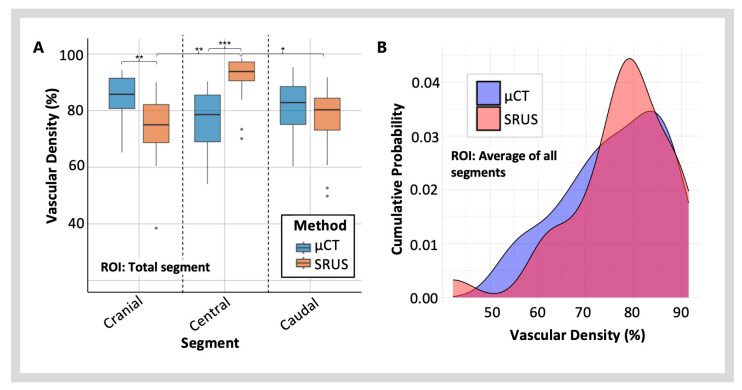
**A comparison between in vivo SRUS and ex vivo µCT vascular density measurements for kidney segments using** (**A**) **ANOVA and** (**B**) **Kolmogorov–Smirnov test of distribution**. The significance values displayed in (**A**) are limited to pair-wise differences between SRUS regions and matched regional data for SRUS and µCT. The appropriate asterisks show the *p*-value, one asterisk (*) for *p* < 0.05, two asterisks (**) for *p* < 0.01, and three asterisks (***) for *p* < 0.001.

Ultrasound attenuation is known to affect MB detection rates [28,29]. To investigate this, the SRUS vascular density measurements in the central segment, within the ultrasound near field, were compared to the two far-field segments, the cranial and the caudal segments (Figure 7A). ANOVA analyses with Tukey’s multiple comparisons analysed vascular density differences between specific segments. The measured vascular density differed significantly between the three segments (*F*(2,84) = 25.86, *p* = 1.77 × 10^−9^ ***).

**Figure 8:**
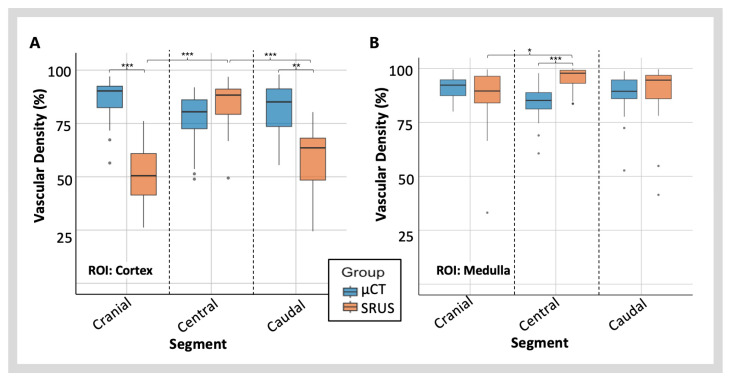
**A comparison between in vivo SRUS and ex vivo µCT regional vascular density measurements for** (**A**) **the cortex and** (**B**) **the medulla.** The significance values displayed represent pairwise comparisons from a two-way repeated-measures ANOVA, which compares SRUS regions to each other and matched segment data from SRUS and µCT. The appropriate asterisks show the *p*-value, one asterisk (*) for *p* < 0.05, two asterisks (**) for *p* < 0.01, and three asterisks (***) for *p* < 0.001.

The central segment had a significantly higher vascular density compared to the cranial (95% CI: 13.75 to 28.84, *p* = 5.84 × 10^−9^ ***) and caudal (95% CI: 10.00 to 25.09, *p* = 9.75 × 10^−7^ ***) segments. There was no significant difference in vascular density between the cranial and caudal segments (*p* = 0.464) (Figure 7A). On further analysis of smaller anatomical regions within these segments, the same pattern of peripheral attenuation on vascular density was seen in the cortex but not in the medulla (Figure 8). In the cortical region, a significantly higher vascular density was seen in the central segment, compared to the cranial (95% CI: 26.90 to 42.62, *p* = 1.58 × 10^−10^ ***) and caudal segments (95% CI: 18.48 to 34.20, *p* = 1.77 × 10^−10^ ***) (Figure 8A). In the medulla, only the cranial segment showed a significant reduction in vascular density compared to the central segment (95% CI: 1.65 to 16.00, *p* = 0.0119 *) (Figure 8B).

#### 3.3.2. Vascular Density Measured with SRUS vs. µCT

To compare the vascular density data from the two imaging modalities, a two-way repeated-measures ANOVA was used, with two factors for the imaging method (µCT vs. SRUS) and three factors for the kidney segment (Figure 7A). In this analysis, the main effect of the imaging method was not significant (*p* = 0.425), indicating no overall difference in vascular density between µCT and SRUS when considering all regions collectively. However, the main effect of the segment was significant (*F*(2,168) = 4.187, *p* = 0.0168 *), showing that vascular density varied across the three kidney regions. The interaction effect between the imaging method and the segment imaged was also significant (*F*(2,168) = 29.598, *p* = 9.73 × 10^−12^ ***), indicating that the difference between µCT and SRUS varies across kidney regions. Both the cranial segment (95% CI: −3.44 to −21.19, *p* = 0.0013 **) and the central segment (95% CI: 11.20 to 28.95, *p* = 1.00 × 10^−6^ ***) showed significant pair-wise differences between the two imaging modalities. The SRUS measured a higher vascular density in the central segment than µCT and lower in the cranial segment. Full pair-wise analysis results can be found in Supplementary Data Table S1. These data were also analysed using the Kolmogorov–Smirnov test, to analyse the vascular density distribution of the two imaging methods for the whole kidney imaging plane, using an average from the three segments (Figure 7B). Supporting the ANOVA results, no difference in the vascular density distribution was detected between the two modalities (*D* = 0.207, *p* = 0.572).

When performing the same analysis on the smaller anatomical regions of the cortex, more significant differences between the modalities were identified (Figure 8A). Significant main effects were observed for both the imaging method (*F*(1,168) = 91.00, *p* = 1.66 × 10^−17^ ***) and image segment (*F*(2,168) = 18.41, *p* = 5.92 × 10^−8^ ***). The interaction between method and region was also significant (*F*(2,168) = 53.75, *p* = 9.04 × 10^−19^ ***), suggesting that the impact of the method varies by region. In the cortical region, significant pair-wise differences between the two imaging modalities were seen in both the cranial (95% CI: 45.66 to 27.61, *p* = 2.22 × 10^−16^ ***) and caudal segments (95% CI: 32.22 to 14.17, *p* = 8.84 × 10^−11^ ***). There was no significant difference in the central cortical segments (*p* = 0.106). A different pattern of vascular density variation was seen with the ANOVA analysis for the medullary region data (Figure 8B). This data showed no significant main effect for the imaging method (*p* = 0.0909) or image segment (*p* = 0.740). A significant interactive effect was detected between the imaging method and the segment imaged (*F*(2,168) = 9.13, *p* = 1.72 × 10^−4^ ***). On medullary imaging, a significant pairwise interaction was only detected in the central segment (95% CI: 3.52 to 18.43), *p* = 5.10 × 10^−4^ ***).

Pairwise data for analysis between µCT regions can be found in Supplementary Data Table S1.

## 4. Discussion

This study investigated the intrinsic variability of SRUS imaging and how it is affected by clinical conditions in a rat model. The analysis found clear associations between clinical conditions and SRUS data parameters, highlighting the need to control experimental variables to ensure the SRUS dataset is reliable for research applications. We identified several technical limitations that users should consider when utilising SRUS for renal imaging, including limited imaging of large arterial vessels and significant impacts of peripheral signal attenuation. We also present a method of quantifying vascular density, comparing it to a method with similar imaging resolution, ex vivo µCT.

### 4.1. The Impact of Experimental Variables on SRUS Imaging

The analysis showed that clinical variables significantly impact SRUS imaging parameters. In SRUS, images are generated from the MB detection tracks. The accumulation of these tracks creates the final microvasculature SRUS image, from which the vessel density is calculated. This explains the correlation between the SRUS parameters (Figure 4).

While HR had an expected significant positive relationship with MAP, no significant correlation with microbubble detection, track reconstruction, or density measurement was observed. This demonstrates the effectiveness of the motion correction technique employed in this study, compensating effectively for the motion associated with changes in the cardiac cycle frequency. A higher MAP and shorter anaesthesia duration were significantly associated with an increase in the number of MB detections and tracks. Even though the strength of the correlations was moderate, both the MAP and anaesthesia duration correlated more strongly with the number of vascular tracks than with MB detection (Figure 4 and Figure 5), likely explained by MB detection being more susceptible to signal noise. Anaesthesia time, but not MAP or HR, was significantly correlated with the SRUS vascular density measurement, whereas no correlation was found with the µCT vascular density measurement (Figure 5F). This linear relationship suggests that anaesthesia duration affects SRUS data collection parameters to a degree, which introduces variability into the accuracy of the vascular density measurement. We explored this further using multiple linear modelling of the SRUS vascular density measurement, which revealed that neither the MAP nor anaesthesia duration independently affected vascular density, but together, they accounted for 45% of the variability. HR had no detectable influence on the variation in SRUS vascular density measurement using linear modelling. We expected to see an association between the MAP and anaesthesia duration due to the hypotensive effects of isoflurane, but there was no significant interaction between the two. Other physiological dynamics must, therefore, be involved. Open abdominal surgery is associated with varying degrees of blood loss and fluid shifts from the intravascular to the interstitial space, both contributing to hypovolemia [30]. Unfortunately, blood loss was not estimated in this study. To compensate, the rats received a continuous infusion of intravenous fluids at 40 μL/min during the experiments. Additionally, prolonged anaesthesia may impair compensatory mechanisms that help maintain homeostasis [27]. The combined effects of these factors likely impact hemodynamic function, including MAP, and may affect MB vascular distribution and signal quality.

We did not anticipate significant SRUS parameter differences across ages (12 to ≈40 weeks) in the Zucker Lean sub-population, which is genetically considered a healthy lab-rat strain. As this rat strain has an average lifespan of 2–3 years, age-related vascular changes would not be expected until beyond one year of age [31]. This was confirmed in this population using µCT; no age-related vascular density changes were observed. However, while no age-related difference in the final SRUS vascular density measurement was detected, higher MB detection rates and more tracks were observed in older animals. We hypothesised that these SRUS parameters may be susceptible to early, age-related changes in MAP and kidney size, but no interaction between MAP and age was observed, nor was there a correlation between kidney size and MB detection.

The significant impacts of the MAP, anaesthesia duration, and age on MB detection and track reconstruction underscore the importance of accounting for physiological variabilities in SRUS imaging to reduce procedural variability and improve reproducibility. The variability in SRUS quality introduced by MAP and anaesthesia likely stems from their impact on hemodynamic conditions, including blood flow dynamics, tissue perfusion, and oxygenation. The broader implications of MAP and physiological state on SRUS reproducibility in clinical settings, particularly in patients with altered hemodynamic function, require further investigation.

### 4.2. Limitations of SRUS Renal Imaging

Previous studies have used MB tracking data from SRUS to estimate blood flow velocity [32,33]. This method is known to underestimate flow velocities significantly [34]. While MBs typically follow the haemodynamics of the vascular system, their movement is also influenced by local vascular architecture, flow conditions, and ultrasound impacts, which together can cause deviations from blood flow velocity [35,36]. MB detection is also innately limited by the frame rate of the imaging system. This study’s 54 Hz frame rate imposed an upper limit of 15 mm/s for detectable MB velocities. Blood flow velocity in the smaller vessels within the rat kidney has not been reliably characterised, but blood flow velocity in the renal vein is estimated at ≈40 mm/s [37] and in the renal artery at ≈700 mm/s [38]. Blood flow velocity decreases as it moves from larger vessels, like arteries, to smaller vessels, such as arterioles and capillaries. Due to the fast arterial flow, blood flow velocity may frequently exceed the limits of the SRUS system, causing MB flow velocities to be significantly underestimated. This study showed that the impact of this MB detection limit varied between vessel types, particularly limiting the imaging of larger arteries with fast flow (Figure 6).

The detection of large arterial vessels was unreliable, with segmental arteries successfully imaged in 34% of the scans. Since segmental arteries and veins run in pairs [39], and segmental veins were visualised in 100% of the scans, the corresponding arteries should also have been detectable. However, because MBs and erythrocytes distribute similarly within vessels [36], we anticipated challenges in imaging large segmental arteries, where blood flow velocities exceed the 15 mm/s detection limit. This contrasts with the consistent detection of segmental veins, where the blood flow velocity is slower. Additionally, the segmental veins exhibited the highest average MB velocities, significantly exceeding those detected in arcuate arteries and arcuate veins, not aligning with the biologically expected patterns of higher arterial blood flow velocities. This discrepancy is most likely caused by the frame rate limitation of the SRUS system, which under-samples faster arterial MBs and over-represents venous MBs within the detectable range.

The smaller arteries, such as the arcuate arteries, were more consistently imaged than the segmental arteries. We hypothesise that the rapid blood flow and MB dispersion over a larger lumen in larger arteries enable many MBs to move at velocities equal to blood velocity, and beyond the detection threshold of 15 mm/s. In contrast, smaller arteries have slower blood flow velocities, and their smaller lumens, branching and tortuosity may slow a more significant proportion of MBs to within the detectable velocity range, thus improving overall detection. This is supported by our finding that lower MAP values were associated with better detection of segmental arteries, suggesting that reduced blood flow velocities under hypotensive conditions improve MB detectability in the arterial system. While the kidney autoregulatory mechanisms aim to stabilise blood flow rates within the microcirculation, beyond the afferent arterioles, larger renal vessels are susceptible to MAP-related changes in blood velocity [40]. The MAP also correlated with estimated blood flow velocities in the veins but not in the arcuate arteries. We hypothesise that most MBs in the arterial system are not detected, as they are moving at a velocity beyond the detection limit of the system. Consequently, the MBs detected in the arterial system are mostly atypical and their velocity is not related to blood haemodynamics. The absence of a linear relationship between arcuate artery MB velocity and MAP, and the biologically impractical finding of arterial velocity being lower than venous velocity, supports this theory.

This study demonstrates that ultrasound attenuation significantly affects SRUS for renal imaging and density analysis (Figure 7 and Figure 8). Vascular density measurements from SRUS were notably higher in the central segment, located within the ultrasound near field, compared to the cranial and caudal far-field segments (Figure 7A). This pattern was not seen in the µCT data. This suggests that the detection of MBs becomes less efficient as the imaging plane moves further from the near field and that this effect is significant enough to impact vascular reconstruction accuracy. This finding is expected, as ultrasound beams are more focused in the near field, leading to higher lateral resolution and the absorption in the tissue reduces the signal-to-noise ratio [41]. A deeper examination of anatomical subregions revealed that this attenuation effect was particularly pronounced in the cortical region, where the central segment showed a significantly higher vascular density than the far-field segments (Figure 8A). These far-field cortical areas represent the most peripheral part of the SRUS image, illustrating how signal strength diminishes with increasing distance from the ultrasound probe, reducing the ability to detect MBs. The attenuation effect was less marked in the medullary region, with less evident regional differences (Figure 8B). This is likely because the medullary regions are located centrally and roughly in the same imaging depth. Additionally, vessels in the medulla, particularly the vasa recta, are straighter and easier to detect, whereas the more tortuous vessels in the cortex, which move in and out of the plane, make MB tracking more challenging.

The Kolmogorov–Smirnov test (Figure 7B) demonstrated a strong agreement between SRUS and µCT for whole-kidney vascular density measurements, confirming similar distribution patterns across datasets from the two modalities. We also detected that the imaging method had no significant main effect on density measurement when analysing full kidneys. However, an analysis of smaller anatomical regions revealed technique-specific variations. This highlights that while the modalities align at a global level, their regional performance varies due to inherent differences in imaging principles and resolution. Across the regions, µCT provided consistent vascular density measurements, with similarities in data spread and range. SRUS measurements exhibited greater variability, especially in the cortical regions, as previously mentioned, due to attenuation, and in the medullary regions, where resolution limitations of the ultrasound system surpassed the diameter of small microvascular vessels, such as the vasa recta (Figure 8). In this study, we estimated a spatial resolution of 42.25 µm using Fourier Ring Correlation. While SRUS has demonstrated potential resolutions down to ≈10 µm, our achieved resolution reflects the limitations of the imaging system, acquisition settings, and biological factors, including imaging depth, in vivo motion, and the acoustic properties of renal tissue. These resolution limitations, combined with the high density and small calibre of medullary vessels, introduce a high degree of inaccuracy in vascular density measurement, resulting in overestimation of vascular density.

### 4.3. Study Limitations

This study has several limitations that should be considered when interpreting the results. One key challenge lies in the inability to directly compare exact density values between SRUS and µCT due to fundamental differences in experimental approaches. Variations such as in vivo versus ex vivo conditions, kidney preparation techniques, and imaging protocols contribute to inconsistencies, making absolute density comparisons unreliable. Ex vivo µCT serves as a valuable tool for identifying trends and visualising vasculature. However, it is important to acknowledge that the appearance of vessels in µCT may not perfectly align with their true anatomical structure. Contrast agent artefacts, such as dilated veins, could lead to overestimating vascular density, while irregular contrast filling and the inability to resolve smaller vessels may result in underestimations. These vascular distortions, known to occur with Microfil [42,43], may account for the limited imaging of segmental arteries with µCT. Another important consideration is the imaging plane used for both methods. In this study, a 2D imaging plane was derived from a 2 mm thick slice of the kidney, and µCT images were processed using maximum intensity projection. This approach inherently overestimates density values compared to a true 3D image of the entire kidney. Nonetheless, the density values remain comparable within this context because the same slice was used for both µCT and SRUS.

Another limitation is the ultrasound data acquisition. A modified BK Medical ultrasound scanner was employed with an amplitude modulation scheme. Three focused emissions in a single direction were used for the amplitude modulation interleaved with B-mode emissions [26]. This rather complicated sequence yielded a low frame rate of 54 Hz, and a low signal-to-noise ratio for the contrast agent. Although data acquisition was not optimal, it yielded 10 min of continuous acquisition, which was not previously possible at the time. Ideally, more advanced parallel imaging sequences should be used, e.g., spherical waves for low mechanical index imaging [44] and scanners developed for long acquisitions [45]. This can give data acquisitions with hundreds to thousands of frames per second for data continuous in time, enhancing signal-to-noise ratio and MB detection and improving MB tracking.

Importantly, there is no consensus on the optimal technical and experimental settings for SRUS imaging. These include factors such as the scanning sequence and acquisition settings, choice of MB injection, and image processing, including the tracking algorithm, thresholding, and motion estimation. Consequently, these specifications vary among research groups, and the optimal settings may differ depending on the tissue type or organ being studied. Therefore, our findings are limited to the technical parameters described here and should not be generalised to the technique.

Finally, broader limitations are associated with the study design, including limited sample size and variability within the study populations. There are also multiple methodological limitations in this study, including the accuracy of manual co-registration, operator bias and accuracy in thresholding selection, and bias in vascular density calculation methods. An objective error evaluation of the co-registration method was not feasible due to the cross-modality and cross-dimensional alignment between 3D μCT data and 2D SRUS images. The co-registration relied on clearly defined anatomical landmarks to guide alignment. Consequently, these same landmarks could not be independently reused for unbiased error assessment, as doing so would conflate the registration process with its evaluation. These constraints should be carefully considered when generalising the findings or drawing conclusions from the data.

There are several limitations related to the clinical translation of our SRUS findings. The vascular physiology and anatomy of rats differ from those of humans, including variations in vessel size, heart rate, and blood flow dynamics. These factors may affect imaging outcomes and must be considered when translating our findings to clinical practice. Further validation in larger animal models and humans is necessary. However, the use of rats in this study enabled a detailed exploration of vascular structures under controlled conditions. These results contribute to the optimisation of imaging protocols and the ongoing adaptation of SRUS for clinical use. Additionally, the ex vivo µCT setup used in this study is not directly translatable to clinical practice.

## 5. Conclusions

This study describes numerous considerations which should be considered when optimising SRUS for clinical and research settings. The data provide evidence of the impact of physiological variables on SRUS imaging in rats, and further research is needed to validate whether the findings are consistent in humans. Like any medical imaging technique, SRUS has inherent limitations, including ultrasound signal attenuation and reduced quantification accuracy when imaging regions with high vascular density and fast blood flow velocities. By comparing in vivo SRUS with ex vivo µCT, we have presented a comprehensive overview of the bias and limits of SRUS renal imaging, as well as a method of quantitative image analysis. Although whole-organ SRUS microvascular imaging requires further technical advancements to address its current limitations, careful selection of target imaging regions and strict control of experimental parameters can enable reproducible and reliable vascular imaging, supporting its utility in research and clinical settings.

## Data Availability

The data used in this paper can be accessed at the following repository: 10.6084/m9.figshare.28485227.

## Supplementary Materials

The following supporting information can be downloaded at https://www.mdpi.com/article/10.3390/diagnostics15121515/s1: Table S1: An overview of pairwise ANOVA results for vascular density.

## Abbreviations

The following abbreviations are used in this manuscript:

SRUS	Super-resolution ultrasound
MB	Microbubble
µCT	Micro-computed tomography
MAP	Mean arterial blood pressure
ROI	Region of interest
2D	Two-dimensional
3D	Three-dimensional
ANOVA	Analysis of variance
ANCOVA	Analysis of covariance
