# Supplementary material for: Analysing the Renal Vasculature Using Super-Resolution Ultrasound Imaging: Considerations for Clinical and Research Applications
Source: Diagnostics (Basel). 2025 Jun 14;15(12):1515. doi: 10.3390/diagnostics15121515 (PMC12192058; doi:10.3390/diagnostics15121515)
Supplement: Supplementary file 1 [file diagnostics-15-01515-s001.zip › diagnostics-3651044-supplementary.pdf]

diagnostics MDPI

Supplementary Materials

Table S1. An overview of pairwise ANOVA results for vascular density. Segment 1 represents the cranial segment, segment 2 the central segment, and segment 3 the caudal segment. The table includes mean difference, 95 % confidence intervals and p-values for pairwise comparisons of vascular density in the following regions: the full segment, cortex and medulla.

Comparison	Region	Mean Difference	95 % CI	P-value
µCT 2: µCT 1	Full segment	-11.10	-19.98 - -2.22	**0.005****
	Cortex	-9.98	-19.01 - -0.95	**0.021***
	Medulla	-6.59	-14.04 - 0.86	0.116
µCT 3: µCT 1	Full segment	-5.06	-13.94 - 3.81	0.570
	Cortex	-5.02	-14.05 - 4.00	0.597
	Medulla	-2.96	-10.41 - 4.50	0.862
µCT 3: µCT 2	Full segment	6.04	-2.84 - 14.91	0.369
	Cortex	4.96	-4.07 - 13.98	0.611
	Medulla	3.63	-3.82 - 11.08	0.723
SRUS 2: SRUS 1	Full segment	21.30	12.42 - 30.17	**$1.38x10^{-09}$*****
	Cortex	34.76	25.73 - 43.78	**$9.55x10^{-15}$*****
	Medulla	8.82	1.37 - 16.28	**0.010***
SRUS 3: SRUS 1	Full segment	3.75	-5.12 - 12.63	0.827
	Cortex	8.41	-0.61 - 17.44	0.083
	Medulla	2.56	-4.89 -10.01	0.920
SRUS 3: SRUS 2	Full segment	-17.54	-26.42 - -8.67	**$7.89x10^{-07}$*****
	Cortex	-26.34	-35.37 - -17.32	**$2.86x10^{-13}$*****
	Medulla	-6.26	-13.71 - 1.19	0.154
SRUS 1: µCT 1	Full segment	-12.32	-21.19 - -3.44	**0.001****
	Cortex	-36.63	-45.66 - -27.61	**$2.22x10^{-16}$*****
	Medulla	-4.44	-11.89 - 3.01	0.522
SRUS 2: µCT 2	Full segment	20.08	11.20 - 28.95	**$1.17x10^{-08}$*****
	Cortex	8.10	-0.93 - 17.13	0.106
	Medulla	10.97	3.52 - 18.43	**$5.08x10^{-04}$*****
SRUS 3: µCT 3	Full segment	-3.50	-12.38 - 5.38	0.865
	Cortex	-23.20	-32.22 - -14.17	**$8.84x10^{-11}$*****
	Medulla	1.08	-6.37 - 8.53	0.998
